# Association between trial registration and treatment effect estimates: a meta-epidemiological study

**DOI:** 10.1186/s12916-016-0639-x

**Published:** 2016-07-04

**Authors:** Agnès Dechartres, Philippe Ravaud, Ignacio Atal, Carolina Riveros, Isabelle Boutron

**Affiliations:** Centre de Recherche Epidémiologie et Statistique, INSERM U1153, Hôpital Hôtel-Dieu, 1 place du Parvis Notre Dame, 75004 Paris, France; Centre d’Epidémiologie Clinique, Hôpital Hôtel-Dieu, Assistance Publique-Hôpitaux de Paris, Paris, France; Faculté de Médecine, Université Paris Descartes, Sorbonne Paris Cité, Paris, France; French Cochrane Centre, Paris, France; Department of Epidemiology, Mailman School of Public Health, Columbia University, New York, USA

**Keywords:** Bias, Registration, ClinicalTrials.gov, Meta-epidemiology, Randomised controlled trial, Meta-analysis

## Abstract

**Background:**

To increase transparency in research, the International Committee of Medical Journal Editors required, in 2005, prospective registration of clinical trials as a condition to publication. However, many trials remain unregistered or retrospectively registered. We aimed to assess the association between trial prospective registration and treatment effect estimates.

**Methods:**

This is a meta-epidemiological study based on all Cochrane reviews published between March 2011 and September 2014 with meta-analyses of a binary outcome including three or more randomised controlled trials published after 2006. We extracted trial general characteristics and results from the Cochrane reviews. For each trial, we searched for registration in the report’s full text, contacted the corresponding author if not reported and searched ClinicalTrials.gov and the International Clinical Trials Registry Platform in case of no response. We classified each trial as prospectively registered (i.e. registered before the start date); retrospectively registered, distinguishing trials registered before and after the primary completion date; and not registered. Treatment effect estimates of prospectively registered and other trials were compared by the ratio of odds ratio (ROR) (ROR <1 indicates larger effects in trials not prospectively registered).

**Results:**

We identified 67 meta-analyses (322 trials). Overall, 225/322 trials (70 %) were registered, 74 (33 %) prospectively and 142 (63 %) retrospectively; 88 were registered before the primary completion date and 54 after. Unregistered or retrospectively registered trials tended to show larger treatment effect estimates than prospectively registered trials (combined ROR = 0.81, 95 % CI 0.65–1.02, based on 32 contributing meta-analyses). Trials unregistered or registered after the primary completion date tended to show larger treatment effect estimates than those registered before this date (combined ROR = 0.84, 95 % CI 0.71–1.01, based on 43 contributing meta-analyses).

**Conclusions:**

Lack of trial prospective registration may be associated with larger treatment effect estimates.

**Electronic supplementary material:**

The online version of this article (doi:10.1186/s12916-016-0639-x) contains supplementary material, which is available to authorized users.

## Background

In 2005, the International Committee of Medical Journal Editors (ICMJE) initiated a policy for trial registration to increase transparency in research. All trials that started recruiting on or after 1 July 2005 should be registered prospectively (i.e. before participant enrolment) as a precondition for publication in member journals [1]. Trials that started recruitment before this date should be registered retrospectively but before 13 September 2005. The World Health Organisation announced its support for trial registration and launched the International Clinical Trials Registry Platform (ICTRP) to facilitate access to existing registries worldwide [2, 3]. These announcements were followed by a massive increase in trial registration that became the norm rather than the exception [3, 4]. However, a substantial proportion of trials remain unregistered. A study published in 2009 found that 28 % of trials published in the 10 general medical and specialty journals with the highest impact factor were not registered [5]. Another found that 39 % of published trials retrieved from MEDLINE appeared not to have been registered [6].

Recently, some researchers generated an important debate among the medical community, arguing that trials published after 2010 that are not prospectively registered should be excluded from Cochrane reviews [7]. Registration aims to make information about the existence and methods of clinical trials publicly available to limit the effect of selective publication of trials and outcomes with positive results resulting in exaggerated treatment effect estimates [8–13]. However, the evidence is as yet unclear concerning a possible association between trial registration and treatment effect estimates [14].

In this study, we aimed to investigate whether there is a difference in treatment effect estimates according to trial prospective registration.

## Methods

We performed a meta-epidemiological study. By using large collections of meta-analyses, these studies are used to assess the association between a trial characteristic and treatment effect estimates [15–18]. For this study, we focussed on Cochrane systematic reviews including randomised controlled trials (RCTs) with results published in 2006 or after. We chose 2006 because registration was required by the ICMJE from September 2005.

### Data sources

We obtained data from all intervention systematic reviews published between March 2011 and September 2014 from the Cochrane Collaboration. Data were provided as XML files and consisted of all elements entered by the review authors in RevMan, the software developed by the Cochrane Collaboration for preparing and maintaining Cochrane reviews. Then, we applied the following selection criteria to perform the meta-epidemiological analyses.

### Study selection

#### Identification of relevant systematic reviews

Using R 3.1.1 with the XML package, we automatically identified all reviews of RCTs with meta-analyses of a binary outcome including three or more RCTs published after 2006. Reviews including observational studies were not considered.

#### Selection of relevant meta-analyses

From the reviews identified, we manually screened all eligible meta-analyses and selected those comparing an active treatment to a placebo or no treatment. Comparisons of two active interventions and meta-analyses of side effects were excluded because of the uncertainty regarding the direction of the bias. If several meta-analyses were eligible per review, we selected, whenever possible, the first meta-analysis including at least four trials (three trials is the minimum to perform meta-epidemiological analyses, four trials allows more power). In case of overlapping meta-analyses across reviews, defined as meta-analyses sharing three or more trials, we selected the one with the largest number of trials, and if they included the same number of trials, we selected the most recent one.

#### Selection of trials

All trials included in the selected meta-analyses were included in the study. RCTs without any events in both arms did not contribute to the analysis.

### Data available from Cochrane reviews

The following data were automatically extracted by using R 3.1.1 with the XML package.

#### Review and meta-analysis characteristics

These characteristics included date of publication, medical condition and, for the selected meta-analysis, interventions being compared and outcome assessed.

#### Trial general characteristics and results

The general characteristics and results included the following:

- Reference of the publication identified from the ʻreferences to studies included in the review’.
- Risk of bias assessment: judgment of bias (i.e. high, low or unclear risk of bias) for each domain of the Cochrane Collaboration Risk of Bias tool and the support for judgment. Because the wording of domains may vary across reviews (e.g. allocation concealment, allocation concealment [selection bias], sequence concealment), we pre-sorted all wording reported in the reviews and manually classified them. According to the Cochrane handbook, the blinding and incomplete outcome data domains should be assessed at the outcome level; therefore, for reviews reporting an evaluation of these domains by outcome or type of outcome, we manually identified the outcome corresponding to the selected meta-analysis.
- Results: for each arm, the number of events as well as the number of patients analysed.

### Trial registration

Then, for each trial, we determined whether it was registered or not using the following sequential approach:

1. We manually searched the full text of each included review for any information regarding registration from the characteristics of included studies and the domains of the Risk of Bias tool: ʻselective outcome reporting’ and ʻother bias’.
2. If no information was reported in the review, we searched for the trial publication abstract and screened whether a registration number was reported.
3. If no information was reported, we searched Google for the publication title with key-words related to registration (i.e. registration, ClinicalTrials.gov, registered, NCT). If there was no result, we retrieved and screened the full text of the publication.
4. For trials for which we were unable to find any information regarding registration, we contacted the corresponding author to ask whether the trial was registered and, if so, in which registry and under which number.
5. In case of no response from the author, two reviewers independently searched ClinicalTrials.gov and the ICTRP using the trial acronym, if any, and keywords concerning population and experimental intervention. All disagreements on trial matching were resolved by consensus. A senior researcher (AD) checked the matching between each trial and registration information.

We classified each trial as (1) prospectively registered, defined as registered before or within a month of the start date (i.e. the date that enrolment to the protocol begins). The ICMJE considers that registration should occur before the start date, whereas the FDA considers that registration should occur within 21 days after the start date. Our definition is in accordance with these statements and takes into account the uncertainty regarding the exact start date, because for most trials, only month and year are reported; (2) retrospectively registered, defined as registered more than 1 month after the start date. We distinguished trials registered before and after the primary completion date (defined as the date of final collection of data for the primary outcome) because we made the assumption that registration after primary completion date could be influenced by the potential knowledge of the results and that it could result in more bias; or (3) not registered.

We also classified trials by compliance with the ICMJE requirements [2]. Trials starting before July 2005 were considered compliant if they were registered before 13 September 2005 and trials starting in July 2005 or later were considered compliant if they were prospectively registered [2].

### Statistical analysis

We estimated treatment outcomes as odds ratios (ORs). Outcome events were re-coded so that an OR <1 indicated a beneficial association with the experimental intervention.

To assess the association between registration and treatment effect estimates, we compared treatment effect estimates between:

1. Prospectively registered and other trials (i.e. unregistered, retrospectively registered and those for which this information was not reported) which is defined as the primary analysis.
2. Trials registered before the primary completion date and registered after or unregistered. Because we had missing data on primary completion date or completion date, we performed two different analyses. In the first analysis, we considered trials for which the primary completion date or completion date was not reported as missing data and did not take them into account in the analysis. In the second analysis, we assumed that trials not reporting the primary completion date were registered after the primary completion date (worst-case scenario).
3. Trials compliant and not compliant with the ICMJE requirement as defined above.
4. Registered and unregistered trials.

For each comparison, we used the following two-step approach described by Sterne et al. [18]. First, for each meta-analysis, we estimated a ratio of odds ratio (ROR) by using a random-effects meta-regression. For the first comparison, for example, this is the ratio of the OR in unregistered or retrospectively registered RCTs to that in prospectively registered RCTs. An ROR <1 indicates larger treatment effect estimates for unregistered or retrospectively registered than prospectively registered trials. Second, we estimated a combined ROR across meta-analyses and the 95 % CI by using a random-effects meta-analysis model. The heterogeneity across meta-analyses was quantified with the I^2^ statistic and the between–meta-analysis variance τ^2^.

All analyses involved the use of Stata SE 11.0 (StataCorp, College Station, TX).

## Results

### Selection and characteristics of systematic reviews

**Fig. 1:**
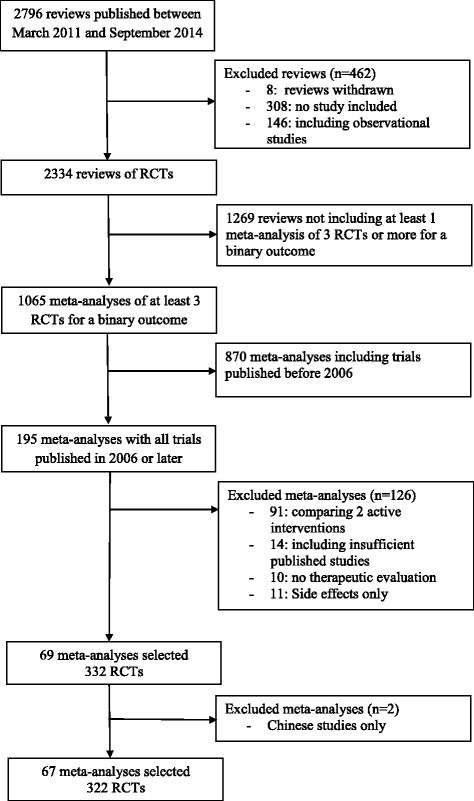
Flow chart of the selection process. *RCT*, randomised controlled trial

The selection process is reported in Fig. 1. Briefly, from 2796 Cochrane reviews published between March 2011 and September 2014, 67 meta-analyses corresponded to our inclusion criteria, for a total of 322 trials. The characteristics of each meta-analysis are reported in the Additional file 1. Briefly, the median number of trials included per meta-analysis was 4 (Q1–Q3: 3–6), with a maximum of 21 trials. The funding source was non-profit in 154 trials (48 %), industry in 104 (32 %) and both non- profit and industry in 22 (7 %).

Overall, 53 meta-analyses assessed a pharmacological intervention for a total of 265 trials and 14 assessed a non-pharmacological intervention for a total of 57 trials.

### Registration of included trials

Information on trial registration was reported in the text of the review for only 17 meta-analyses (25 %), including 7 (10 %) for which this information was systematically reported. Overall, 225/322 trials (70 %) were registered; the median proportion of registered trials per meta-analysis was 71 % (Q1–Q3: 55–100 %). Registration was prospective for 74 trials (33 %) and retrospective for 142 (63 %), with 88 registered before the primary completion date and 54 after. The start date and/or primary completion date was not reported for 9 (4 %) trials. Characteristics of trials by registration status are listed in Table 1. Briefly, among the 133 trials having started in July 2005 or after, 69 (52 %) were prospectively registered, 29 (22 %) were retrospectively registered but before the primary completion date, 11 (8 %) were registered after the primary completion date, 2 were retrospectively registered and did not report the primary completion date and 22 (17 %) were not registered. Thirty-six (23 %) of non-profit trials were prospectively registered as compared with 35 (34 %) of industry-funded trials.

**Table 1:** Characteristics of included trials by registration status

Characteristics	Registered *N* = 225				Not registered *N* = 97
	Prospectively *N* = 74	Retrospectively before PCD^b^ *N* = 88	Retrospectively after PCD^b^ *N* = 54	Not reported *N* = 9	
Type of intervention^a^					
Pharmacological (*N* = 265)	66 (25)	72 (27)	46 (17)	7 (3)	74 (28)
Non-pharmacological (*N* = 57)	8 (14)	16 (28)	8 (14)	2 (4)	23 (40)
Publication year^a^					
2006–2009 (*N* = 169)	17 (10)	53 (31)	31 (18)	2 (1)	66 (39)
2010–2014 (*N* = 153)	57 (37)	35 (23)	23 (15)	7 (5)	31 (20)
Start date^a^					
Before July 2005 (*N* = 162)	5 (3)	59 (36)	41 (25)	6 (4)	51 (31)
In July 2005 or after (*N* = 133)	69 (52)	29 (22)	11 (8)	2 (1)	22 (17)
Not reported (*N* = 27)	0 (0)	0 (0)	2 (7)	1 (4)	24 (89)
Sample size					
Median (Q1–Q3)	242 (100–632)	152 (66–439)	153 (64–274)	97 (63–103)	87 (50–154)
Funding^a^					
Non-profit (*N* = 154)	36 (23)	50 (32)	29 (19)	4 (3)	35 (23)
Industry (*N* = 104)	35 (34)	31 (30)	19 (18)	4 (4)	15 (14)
Both (*N* = 22)	2 (9)	6 (27)	6 (27)	0 (0)	8 (37)
Not reported (*N* = 42)	1 (2)	1 (2)	0 (0)	1 (2)	39 (93)
Risk of bias					
Sequence generation (high/unclear)	19 (26)	17 (19)	16 (30)	5 (56)	27 (28)
Allocation concealment (high/unclear)	25 (34)	26 (29)	21 (39)	6 (67)	47 (48)
Incomplete outcome data (high/unclear)	24 (34)	35 (40)	12 (22)	1 (11)	33 (34)

^a^Percentages are calculated by row

^b^PCD = primary completion date

### Comparison of treatment effect estimates between prospectively registered and unregistered or retrospectively registered trials

**Fig. 2:**
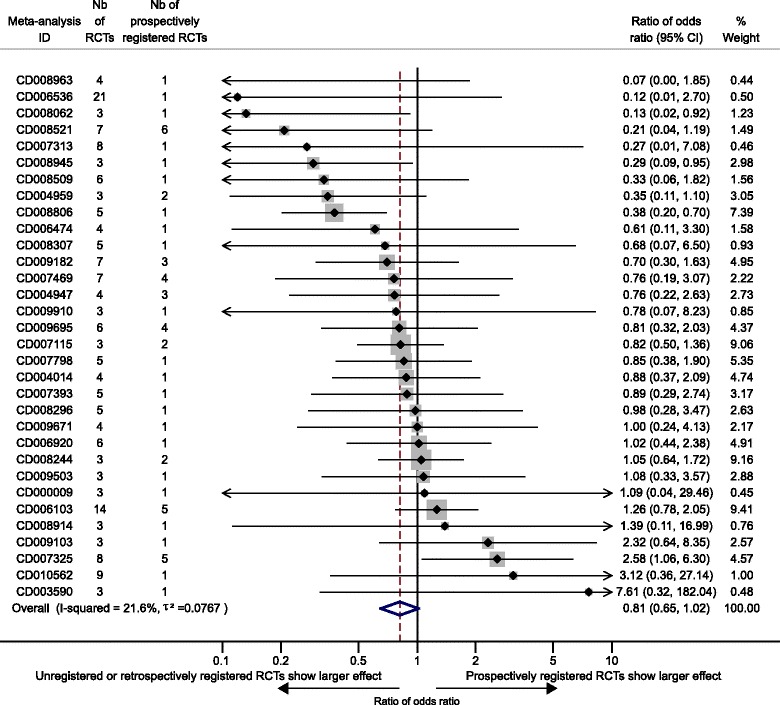
Comparison of treatment effect estimates between unregistered or retrospectively registered and prospectively registered trials. Difference in treatment effect estimates is expressed as ratio of odds ratio (*ROR*). An ROR <1 indicates larger treatment effect estimates in trials retrospectively registered or not registered

From 32 meta-analyses (165 trials), unregistered or retrospectively registered trials tended to show larger treatment effect estimates than prospectively registered trials (combined ROR = 0.81, 95 % CI 0.65–1.02), with low heterogeneity across meta-analyses, I^2^ = 21.6 % and between–meta-analyses variance τ^2^ = 0.0767 (Fig. 2).

### Comparison of treatment effect estimates between trials registered before primary completion date and those registered after or not registered

**Fig. 3:**
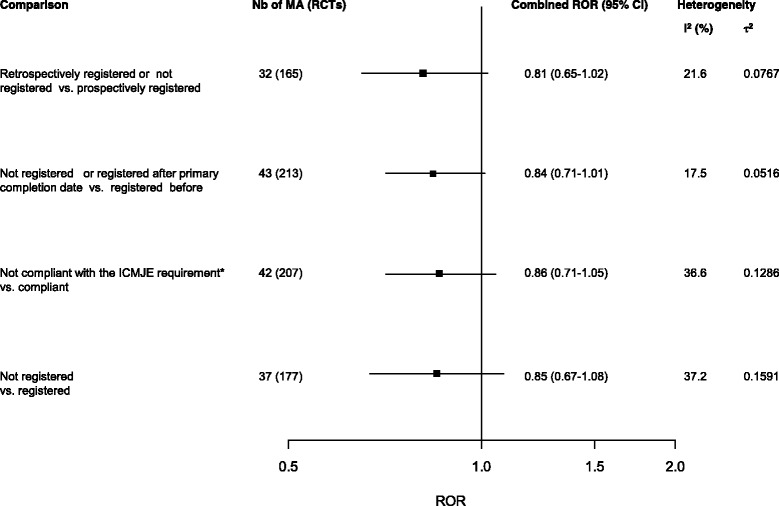
Comparison of treatment effect estimates by trial registration. Difference in treatment effect estimates is expressed as ROR. An ROR <1 indicates larger estimates of treatment effect in trials retrospectively registered or not registered, registered after the primary completion date, not compliant with the ICMJE and not registered, respectively. * Compliance with the ICMJE requirement is defined as registration before 13 September 2005 for trials starting before July 2005 and prospective registration for trials starting in July 2005 or after [2]. *ICMJE* International Committee of Medical Journal Editors

From 43 meta-analyses (213 RCTs), trials registered after the primary completion date or unregistered tended to show larger estimates than those registered before (combined ROR = 0.84, 95 % CI 0.71–1.01), with low heterogeneity across meta-analyses, I^2^ = 17.5 % and τ^2^ = 0.0516 (Fig. 3). A sensitivity analysis considering trials for which the primary completion date was missing as trials registered after the primary completion date gave consistent results (ROR = 0.85, 95 % CI 0.72–1.01, I^2^ = 15.6 %, τ^2^ = 0.0449).

### Comparison of treatment effect estimates between trials compliant and not compliant with the ICMJE requirement

From 42 meta-analyses (207 RCTs), we found a combined ROR = 0.86 (95 % CI 0.71–1.05) with larger estimates in trials not compliant to the ICMJE requirement; heterogeneity across meta-analyses was I^2^ = 36.6 %, τ^2^ = 0.1286 (Fig. 3).

### Comparison of treatment effect estimates between unregistered and registered trials

From 37 meta-analyses (177 trials), the combined ROR between unregistered and registered trials was 0.85 (95 % CI: 0.67–1.08) with I^2^ = 37.2 % and τ^2^ = 0.1591 (Fig. 3).

## Discussion

With this study, we aimed to provide some evidence concerning the association between trial registration and treatment effect estimates. From a sample including all Cochrane reviews with trials published in 2006 or after, our results suggest that trials retrospectively registered or not registered may show larger treatment effect estimates than other trials. All analyses gave consistent results.

This is the first meta-epidemiological study assessing the association between trial registration and treatment effects. Meta-epidemiological studies are considered the gold standard for assessing bias [16]. To determine whether a trial was registered, we used a sequential approach involving contacting corresponding authors and a duplicate search of registries in case of lack of information in trial reports. Because assessing the impact of prospective registration is complex in that it became a requirement for trials starting after July 2005, we conducted several complementary analyses (e.g. registration after the primary completion date, compliance with the ICMJE requirement), all of which gave consistent results with a trend to larger treatment effect estimates for unregistered trials and those retrospectively registered or registered after the primary completion date.

Two previous studies compared the conclusions of trials by registration status in specific medical areas [19, 20]. One found that trials registered before publication and those unregistered were equally likely to reach conclusions favouring new oncology drugs [20]. A more recent study found that trials in cardiology reported as registered were less likely to report positive findings than those not reported as registered [19]. Our results suggest that trial registration is an important element to consider because it may be associated with treatment effect estimates using a meta-epidemiological approach. Results from meta-epidemiological studies were used to determine the items associated with treatment effect estimates that could be related to bias and served as a basis to develop the Cochrane Risk of Bias tool [15, 16]. Meta-epidemiological studies have identified other characteristics not directly associated with a bias-producing process in an individual trial but manifesting when looking at collections of trials. Such characteristics including funding sources, single-centre status or sample size have been considered meta-bias [21]. Trial registration may be another type of meta-bias.

### Limitations

Our study has some limitations. To perform the meta-epidemiological analysis, we had to predefine relatively restrictive selection criteria (i.e. selection of meta-analyses involving three RCTs or more), which resulted in a limited number of meta-analyses that may not be representative of all Cochrane reviews. Our analyses may lack power and we could only observe a trend but no statistically significant results, so our results should be interpreted carefully. We did not perform a formal sample size calculation because this is complex for meta-epidemiological studies and because of the uncertainty regarding the amount of difference in treatment effect estimates by registration status [22]. We used all meta-analyses corresponding to our eligibility criteria from our sample of Cochrane reviews and reported the results transparently. We did not attempt to increase our sample size a posteriori because this would have been driven by our results. Such post hoc decisions are criticised and may result in overestimated associations. Even if we had found a statistical difference, we think that this would not be sufficient to justify decisions regarding the exclusion of trials from Cochrane reviews based on their registration status. This rather highlights the importance of systematically collecting this item and performing sensitivity analyses when conducting meta-analyses to assess whether it could affect the results. The number of meta-analyses and the number of trials per meta-analysis also limit the ability to explore whether meta-confounding by trial funding sources, sample size and risk of bias could explain our results because these characteristics are frequently associated with treatment effect estimates. Industry-sponsored trials are more likely to comply with registration policies than non-profit-funded trials [23–25] and also more likely to show more favourable results [26]. Smaller trials or trials at high risk of bias might be more likely to be retrospectively registered or unregistered. Nevertheless, it has to be noted that most meta-epidemiological studies do not adjust on possible confounding factors [27].

### Implications

A first implication for future research is to confirm our results in larger meta-epidemiological studies that could also allow adjustment for important confounding factors like funding source or sample size.

Other important implications can be discussed. Our results remind us of the importance of prospective registration for all trials which should be systematically verified by peer reviewers and editors during the peer review process. We suggest a careful interpretation of trials not registered or retrospectively registered, particularly those registered after the primary completion date. At the systematic review level, there is a need for systematically collecting and reporting information on trial registration for each included trial, which is currently not frequently done in systematic reviews. Although it is recommended to systematically report this information in reports of RCTs [28, 29], the PRISMA Statement [30, 31] and the Cochrane Handbook [16] do not contain recommendations for collecting and reporting this information when conducting a systematic review. Accounting for trial registration during the meta-analysis process is challenging. Our results cannot allow for recommending the exclusion of trials not registered or retrospectively registered from meta-analyses. Nevertheless, some arguments suggest that this approach is not appropriate, as it may lead to the exclusion of more recent trials and of trials funded by academic sources as they are less likely than industry-funded trials to comply with registration policy [23–25]. We rather recommend that review authors conduct sensitivity analyses based on registration status to check whether it has an influence on the results.

## Conclusions

Our results suggest that trial registration may be associated with treatment effect estimates, with a tendency for larger effects in unregistered or retrospectively registered trials. Our results should be confirmed in other meta-epidemiological studies but highlight the importance of prospective registration for all trials.

## Abbreviations

CI, confidence interval; ICMJE, International Committee of Medical Journal Editors; ICTRP, International Clinical Trials Registry Platform; OR, odds ratio; RCT, randomised controlled trial; ROR, ratio of odds ratio

## Additional file

Additional file 1: Characteristics of included meta-analyses. (DOC 152 kb)
