# Supplementary material for: Association between trial registration and treatment effect estimates: a meta-epidemiological study
Source: BMC Med. 2016 Jul 4;14:100. doi: 10.1186/s12916-016-0639-x (PMC4932748; doi:10.1186/s12916-016-0639-x)
Supplement: Additional file 1: — Characteristics of included meta-analyses. (DOC 152 kb) [file 12916_2016_639_MOESM1_ESM.doc]

**Additional file 1. Appendix 1: Characteristics of included meta-analyses**

| **Cochrane ID** | **Medical condition** | **Interventions** | **Outcome** | **No. of trials** | **No. of registered trials (prospective/retrospective)** | **Overall OR (95% CI)** | **I^2^ (%)** |
| --- | --- | --- | --- | --- | --- | --- | --- |
| CD000009 | Tobacco use | Acupressure vs. sham acupressure | Short term smoking cessation | 3 | 1 (1/0) | 0.33 (0.15-0.74) | 0 |
| CD003410 | Chronic heroin dependency | Supervised injection heroin+methadone vs. methadone | Retention in treatment | 4 | 4 (0/4) | 0.34 (0.27-0.42) | 0 |
| CD003590 | Protection of renal function in the perioperative period | Atrial natriuretic peptide vs. no intervention | All-cause mortality | 3 | 3 (1/2) | 0.61 (0.16-2.37) | 16 |
| CD003617 | Chronic hepatitis C | Interferon vs. control | All-cause mortality | 3 | 2 (0/2) | 1.16 (0.51-2.63) | 44 |
| CD004014 | Pelvic organ prolapsed in women | Use of graft vs. no graft | Objective failure | 4 | 3 (1/2) | 0.56 (0.36-0.87) | 0 |
| CD004136 | Diabetes | Angiotensin receptor blockers vs. placebo or no treatment | Normo- to micro- or macroalbuminuria | 5 | 5 (0/5) | 0.89 (0.64-1.25) | 59 |
| CD004147 | Opioid dependence | Counselling+pharm vs. pharm | Retention in treatment* | 4 | 2 (0/1, 1=nr) | 0.75 (0.53-1.07) | 0 |
| CD004152 | Replacement of missing teeth | Antibiotics vs. placebo or no treatment | Implant failure | 6 | 0 | 0.31 (0.15-0.65) | 0 |
| CD004415 | Actinic keratoses | Ingenol mebutate (PEP005) vs. placebo | Cosmetic outcome: changes in pigmentation | 3 | 3 (3/0) | 3.98 (0.65-24.3) | 16 |
| CD004734 | Unexplained recurrent miscarriage | Low molecular weight heparin vs. no treatment | Live birth | 3 | 1 (0/1) | 0.33 (0.16-0.68) | 38 |
| CD004947 | Short cervix leading to risk of preterm birth (singletons) | Progesterone vs. placebo | Respiratory distress syndrome | 4 | 4 (3/1) | 0.68 (0.43-1.06) | 20 |
| CD004959 | Chronic low-back pain | Strong opioids vs. placebo | Pain relief (at least 30% or moderate relief) | 3 | 3 (2/1) | 0.47 (0.27-0.82) | 38 |
| CD005346 | Acute ischaemic stroke | Insulin therapy vs. usual care | Death | 9 | 4 (2/2) | 1.09 (0.85-1.41) | 0 |
| CD006086 | Tuberculosis | Zinc+vitamin A vs. placebo | Death by 6 months | 7 | 5 (0/2, 3=nr) | 2.22 (0.74-6.63) | 0 |
| CD006103 | Tobacco use | Varenicline vs. placebo | Continuous or sustained abstinence at longest follow-up | 14 | 12 (5/7) | 0.36 (0.29-0.45) | 54 |
| CD006185 | Stroke | Electromechanical and robotic-assisted gait training plus physiotherapy vs.  physiotherapy | Recovery of independent walking | 5 | 2 (0/2) | 0.32 (0.18-0.57) | 17 |
| CD006423 | Type 2 diabetes mellitus | Liraglutide 1.8 mg vs. placebo | HbA1c < 7% | 4 | 4 (4/0) | 0.19 (0.12-0.31) | 63 |
| CD006458 | Acute bronchiolitis in infants | Hypertonic saline versus normal saline | Re-admission | 3 | 2 (0/2) | 1.06 (0.58-1.93) | 0 |
| CD006474 | Prevention of allergy in infants | Prebiotic vs. no prebiotic | Eczema | 4 | 1 (1/0) | 0.70 (0.40-1.21) | 37 |
| CD006536 | Acute myocardial infarction | Stem cells vs. no stem cells | Mortality | 21 | 15 (1/13, 1=nr) | 0.69 (0.38-1.27) | 0 |
| CD006780 | Risk of placental dysfunction | Heparin vs. no treatment | Preterm birth less than 34 weeks' gestation | 3 | 2 (0/2) | 0.39 (0.23-0.68) | 0 |
| CD006895 | Prevention of acute upper respiratory tract infections | Probiotics vs. placebo | No. of participants who used antibiotics | 3 | 2 (0/2) | 0.59 (0.36-0.96) | 0 |
| CD006920 | Subfertility | Acupuncture vs. control | Miscarriage | 6 | 2 (1/1) | 1.11 (0.73-1.68) | 0 |
| CD007115 | Pain in major depressive disorder | Duloxetine vs. placebo | > 30% pain relief at 12 weeks or less | 3 | 3 (2/1) | 0.60 (0.49-0.75) | 0 |
| CD007189 | High risk of HIV infection | Antiviral pre-exposure prophylaxis vs. placebo | HIV infection | 4 | 4 (4/0) | 0.48 (0.27-0.85) | 76 |
| CD007235 | Prevention of preterm delivery | Cervical assessment vs usual care | Preterm birth < 34 weeks | 3 | 2 (0/2) | 0.52 (0.22-1.23) | 0 |
| CD007313 | Chronic asthma | Single inhaler combined therapy vs. usual care | Patients with exacerbations causing hospitalisation | 8 | 8 (1/7) | 0.85 (0.48-1.52) | 0 |
| CD007325 | Macular oedema secondary to central retinal vein occlusion | Anti-vascular endothelial growth factor vs. sham injection | Gain of ≥ 15 letters at 6 months | 8 | 8 (5/3) | 0.22 (0.15-0.32) | 21 |
| CD007393 | Chronic neuropathic pain | Topical capsaicin vs. control | At least 50% pain intensity reduction over weeks 2 to 8 | 5 | 5 (1/0, 4=nr) | 0.63 (0.45-0.88) | 0 |
| CD007400 | Chronic musculoskeletal pain | Topical NSAID vs. placebo | Clinical success | 4 | 4 (0/3, 1=nr) | 0.67 (0.57-0.78) | 0 |
| CD007469 | Prevention of cancer | Vitamin D vs. placebo or no intervention | Breast cancer occurence | 7 | 7 (4/2, 1=nr) | 0.97 (0.86-1.09) | 0 |
| CD007798 | Peripheral venous catheters | Clinically indicated replacement vs. routine care | Phlebitis | 5 | 4 (1/3) | 1.16 (0.93-1.45) | 0 |
| CD008062 | Skin preparation prior to surgery | Microbial sealant vs. no microbial sealant | Surgical site infection | 3 | 2 (1/0, 1=nr) | 0.24 (0.05-1.20) | 54 |
| CD008079 | Chronic lymphocytic leukemia | Anti-leukaemic therapy plus anti-CD20 vs. anti-leukaemic therapy alone | Overall response rate | 3 | 3 (0/3) | 0.54 (0.42-0.70) | 3 |
| CD008244 | Fibromyalgia | Milnacipran 100 mg/day vs. placebo | Composite 1: ≥ 30% pain relief + PGIC much or very much improved | 3 | 2 (2/0) | 0.61 (0.50-0.75) | 0 |
| CD008296 | Schizophrenia | Paliperidone palmitate vs. placebo | Relapse: recurrence of psychotic symptoms | 5 | 5 (1/4) | 0.51 (0.33-0.79) | 63 |
| CD008307 | Prevention of chronic pain after surgery | Ketamine vs. placebo | Incidence of any pain at 3 months | 5 | 2 (1/1) | 0.72 (0.43-1.20) | 0 |
| CD008367 | Critical care | Toothbrushing vs. no toothbrushing | Incidence of ventilator-assisted pneumonia | 4 | 4 (0/4) | 0.69 (0.36-1.29) | 64 |
| CD008386 | Multiple sclerosis | Statins + beta interferon vs. beta interferon | Relapses | 3 | 1 (0/1) | 1.13 (0.59-2.15) | 18 |
| CD008409 | Acute traumatic brain injury | Progesterone vs. placebo | Mortality | 3 | 2 (0/2) | 0.53 (0.28-1.01) | 20 |
| CD008472 | Vascular and endovascular surgical procedures | Remote ischaemic preconditioning vs. usual care | Perioperative mortality | 3 | 3 (0/3) | 1.50 (0.36-6.35) | 2 |
| CD008509 | Ureteral stones | Alpha blockers vs. placebo | Stone clearance | 6 | 3 (1/2) | 0.48 (0.26-0.88) | 62 |
| CD008521 | Prevention of rotavirus diarrhoea | Vaccine vs. placebo | Severe rotavirus diarrhoea | 7 | 7 (6/1) | 0.44 (0.29-0.68) | 58 |
| CD008716 | Hepatic encephalopathy | Probiotics vs. placebo or no intervention | Change of or withdrawal from treatment | 3 | 1 (0/1) | 1.33 (0.47-3.72) | 0 |
| CD008806 | Acute bacterial meningitis | Glycerol vs. no osmotic therapy in addition to usual care | Seizures | 5 | 1 (1/0) | 1.08 (0.63-1.83) | 64 |
| CD008834 | Chronic kidney disease | Antiplatelet agent vs. control | All-cause hospitalization | 3 | 2 (0/2) | 0.93 (0.78-1.12) | 0 |
| CD008907 | Drug-resistant partial epilepsy | Eslicarbazepine acetate 800 mg/day vs. placebo | Seizure free | 3 | 3 (0/3) | 0.26 (0.08-0.81) | 0 |
| CD008914 | Pancreaticoduodenectomy | Stent vs. no stent | Pancreatic fistulas | 3 | 2 (1/1) | 0.62 (0.26-1.50) | 73 |
| CD008945 | Chronic pancreatitis | Antioxydants vs. control | Pain free | 3 | 2 (1/1) | 0.38 (0.17-0.89) | 55 |
| CD008959 | Nutrition in children under 2 years of age | Provision of multiple micronutrient powders vs. no intervention or placebo | Anaemia | 6 | 1 (0/1) | 0.44 (0.32-0.61) | 41 |
| CD008963 | Osteoarthritis | Self management programme vs. information only | Withdrawals | 4 | 3 (1/2) | 1.98 (0.64-6.18) | 87 |
| CD009103 | Secondary prevention of stroke | Educational or behavioural interventions for patients vs. usual care | Blood pressure target achievement | 3 | 2 (1/0, 1=nr) | 0.74 (0.39-1.44) | 35 |
| CD009154 | Subfertility | Progesterone vs. progesterone + estrogen | Miscarriage | 7 | 1 (0/1) | 1.01 (0.60-1.69) | 30 |
| CD009182 | Chronic hepatitis C | Nitazoxanide vs. placebo or no intervention | Failure of sustained virological response | 7 | 5 (3/2) | 0.64 (0.42-0.97) | 0 |
| CD009318 | Neuropathic pain and fibromyalgia | Lacosamide 400 mg versus placebo | Moderate benefit | 4 | 4 (0/4) | 0.61 (0.45-0.83) | 0 |
| CD009363 | Fractures of the middle third of the clavicle | Surgical vs. conservative interventions | Overall treatment failure | 7 | 1 (0/1) | 0.35 (0.11-1.10) | 32 |
| CD009452 | Assisted reproduction | Heparin vs. usual care | Live birth | 3 | 2 (0/1, 1=nr) | 0.55 (0.24-1.23) | 49 |
| CD009503 | Acute myocardial infarction | Adenosine and verapamil vs. usual care | Myocardial blush grade 0 to 1 after primary percutaneous coronary intervention | 3 | 2 (1/1) | 0.95 (0.67-1.34) | 0 |
| CD009542 | Milk allergy | Milk oral immunotherapy vs. control | Full desensitization | 5 | 3 (0/2, 1=nr) | 0.03 (0.01-0.09) | 0 |
| CD009671 | Primary prevention | Selenium supplementation vs. no intervention | Type 2 diabetes | 4 | 3 (1/2) | 1.14 (0.83-1.58) | 37 |
| CD009695 | Amphetamine dependence | Psychostimulants vs. placebo | Sustained abstinence | 6 | 6 (4/2) | 0.78 (0.50-1.20) | 0 |
| CD009764 | Chronic obstructive pulmonary disease | Antibiotics vs. placebo | One or more exacerbations | 4 | 4 (0/3, 1=nr) | 0.64 (0.45-0.90) | 62 |
| CD009910 | Moderate or severe persistent asthma | Bronchial thermoplasty | Hospital admission | 3 | 3 (1/2) | 3.81 (1.33-10.97) | 0 |
| CD010210 | Acute postoperative pain | Ibuprofen 200 mg + paracetamol 500 mg vs. placebo | Participants with at least 50% pain relief | 3 | 3 (0/3) | 0.03 (0.02-0.07) | 0 |
| CD010253 | Liver transplantation | T-cell antibody induction vs placebo/no intervention | Diabetes mellitus | 4 | 0 | 0.86 (0.61-1.22) | 0 |
| CD010562 | Surgery | Topical tranexamic acid vs. control | Deep vein thombosis | 9 | 6 (1/5) | 0.69 (0.22-2.17) | 15 |
| CD010943 | Neuropathic pain | Levetiriacetam versus placebo | Subtantial pain relief (at least 50% or complete or good pain relief) | 4 | 4 (2/1, 1=nr) | 1.22 (0.50-2.94) | 0 |
